# Hydrophilic Magnetochromatic Nanoparticles with Controllable Sizes and Super-high Magnetization for Visualization of Magnetic Field Intensity

**DOI:** 10.1038/srep17063

**Published:** 2015-11-23

**Authors:** Lin Zhuang, Yongxin Zhao, Huixiang Zhong, Jinhua Liang, Jianhua Zhou, Hui Shen

**Affiliations:** 1 School of Physics and Engineering, State Key Laboratory of Optoelectronic Materials and Technologies, Sun Yat-sen University, Guangzhou 510006, China; 2 Key Laboratory of Sensing Technology and Biomedical Instruments of Guangdong Province, School of Engineering, Sun Yat-sen University, Guangzhou 510006, China

## Abstract

Hydrophilic Fe_3_O_4_ nanoparticles with controllable size and shape have been fabricated using a facile solvothermal approach followed by surface modification with polyacrylic acid (PAA). The nanoparticles form one-dimension photonic crystal structure under external magnetic field ranging from 29.6 to 459 G. The reflection peaks of formed photonic crystals cover the entire visible spectrum, which indicates a good magnetochromatic property and prospect of wide applications. The size and shape of Fe_3_O_4_ nanoparticles are controlled by changing the ratio between ethylene glycol and diethylene glycol. In the process of surface modification, PAA synthesized by free radical polymerization was chemisorbed onto the surface of Fe_3_O_4_ particles with the aid of Fe^3+^ cations, which renders the particles well dispersed in aqueous solution with high thermo-stability. The Fe_3_O_4_ particles exhibit ferrimagnetism with a high saturation magnetization value of 88.0 emu/g. Both the high magnetization and the wide reflection spectrum under magnetic field make the magnetochromatic nanoparticles a promising material for visualization of the distribution of magnetic field intensity on microfluidic chips.

Hydrophilic Fe_3_O_4_ nanoparticles (FNPs) have attracted a growing research interest in their potential applications including advanced magnetic devices, display, and medical diagnostic^1^,^2^,^3^,^4^,^5^. Color-tunable microactuator utilizing optical and magnetic behaviors of FNPs has been realized^6^. Structural color patterning were also demonstrated by using the magnetochromatic characteristics of FNPs and microspheres^7^. The magneto-optical properties of the FNPs, including birefringence^8^,^9^, optical transmittance^10^,^11^,^12^ and magnetochromatics^13^, were widely investigated, and it is found that the magnetic and magneto-optical properties are heavily dependent on the size, crystal structure and the surface modification of the nanoparticles.

Controlled synthesis of FNPs with expected size, morphology and surface properties have been studied by many groups. Methods including coprecipitaion of ferrous and ferric ions^14^,^15^, high-temperature hydrolysis^16^,^17^, hydrothermal^18^, solvothermal^19^,^20^,^21^ and thermal decomposition processes^22^,^23^ have been reported, and attempts for improving these methods are still being explored. Cui *et al.* utilized a one-pot method to prepare hydrophilic FNPs soluble in water in the presence of citrate and sodium nitrate, FNPs is formed from the dehydration reaction of ferrous hydroxide and ferric hydroxide, and their size distribution are between 20 to 40 nm^24^. Yin and his co-workers reported a high-temperature hydrolysis method to grow monodisperse superparamagnetic FNPs with well-controlled size and shape; the saturation magnetization for the particles with the size of 174 nm was 63.5 emu/g. The size of the particles were tuned by the amount of NaOH/diethylene glycol solution, which makes them susceptible to the alkalinity of the system^16^. Li *et al.* first reported the high saturation magnetization of monodisperse FNPs which was 81.9 emu/g for the as-prepared 200 nm nanopheres; their high yields, inexpensive synthesis process was desirable for wide application. However, such Fe_3_O_4_ microspheres were difficult to be dispersed in aqueous media due to their large size and strong magnetic interaction^20^. A facile strategy for synthesizing size-controllable and stable FNPs with high magnetization is still worthy of exploring. Many efforts for visualization of the magnetic field distribution have been attempted. Thin films using the magneto-optical properties were reported to visualize the magnetic fluxes although how to avoid scratches of the films on substrate has still to be solved^25^,^26^. Computer tomography method was studied for magnetic measurements which is represented by vector values^27^. Magneto-optical imaging system was also used to intuitively observe the magnetic field distribution^28^. These methods need complex and time consuming procedures to obtain a complete map of the field distribution. To the best of our knowledge, by using of the nanoparticles with magnetochromatic property and directly observing the color changes of the particles solution to determine the magnetic field intensity has hardly been reported.

Here, we report an approach to prepare FNPs with enhanced hydrophilicity and controlled sizes and morphology. The particles well dispersed in water exhibits outstanding magnetic and magnetochromatic properties, which could be used for visualization of magnetic field intensity. We precisely controlled the sizes and shapes of the particles by changing the ratio of diethylene glycol (DEG) and ethylene glycol (EG). We modified the FNPs with PAA, enabling the particles to disperse in aqueous media with relatively high ionic strength. The particles solution shows good thermo-stability, as well as keeping high magnetic saturation (81.5, 88.0 emu/g for the particles with 60, 100 nm respectively). The particles form one-dimension photonic crystal structure under external magnetic field and reflect visible light ranging from 480 ~ 680 nm. The potential application of these hydrophilic Fe_3_O_4_ particles for measuring the distribution of magnetic field intensity on a chip is demonstrated, and the magnetic field intensity varying from 114 to 421 G can be visualized/imaged under an optical camera. These FNPs are thus promising candidates for the applications of the magnetic field intensity measurement, inexpensive structural color patterning, and color display devices, etc.

## Results and Discussion

### The shapes and sizes of the as-prepared Fe_3_O_4_ nanoparticles

**Figure 1:**
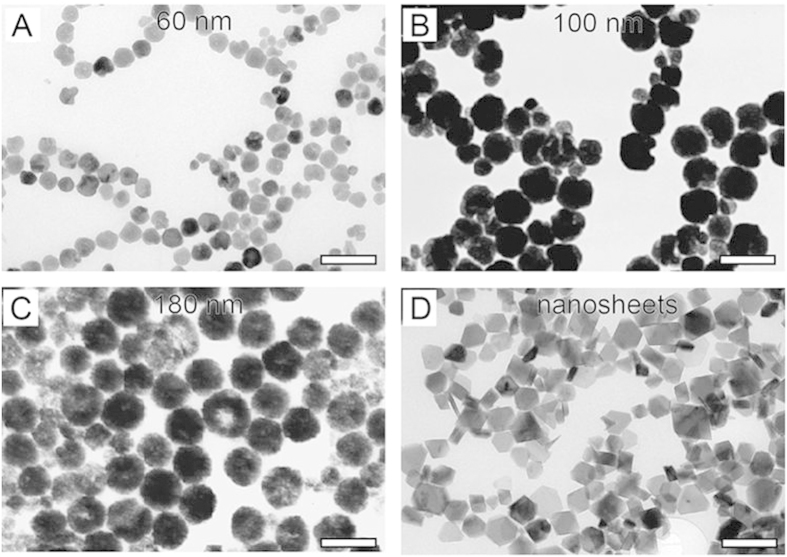
TEM images of (A) 60 nm Fe_3_O_4_ spherical particles, (B) 100 nm Fe_3_O_4_ spherical particles, (C) 180 nm Fe_3_O_4_ spherical particles, and (D) Fe_3_O_4_ nanosheets. The scale bars are 200 nm.

Figure 1 shows the TEM images of the as-prepared modified Fe_3_O_4_ nanoparticles and nanosheets. Clearly, in the case of the DEG/EG volume ratios of 0/40, 26/14, 30/10 and 40/0, the spherical FNPs with the diameter of 180 nm, 100 nm, and 60 nm and the Fe_3_O_4_ nanosheets (~90 nm) are obtained respectively. The size distributions of these FNPs are narrow, with a coefficient of 0.08. It can be known that the volume ratio of DEG to EG plays an important role in the formation of the size and shape of the FNPs. The X-ray diffraction patterns of the as-prepared Fe_3_O_4_ nanoparticles and nanosheets synthesized with different DEG/EG volume ratios are shown in Figure 1S, which reveal the formation of magnetite iron oxide with well-defined crystallinity.

Compared with the reported synthesis methods, our preparation approach has several advantages. First, the surface modification of the Fe_3_O_4_ particles in our method was performed after the synthesis process of the particles. Obviously, the stabilizer is not involved in the synthesis process of magnetic particles, which simplifies the synthesis process and increases the reproducibility. Second, due to the reducing environment created by EG and DEG, inert gas protection is not necessary in the closed system, which makes our approach much simpler. Also, the shape and size of the particles are precisely controlled by the ratio of the DEG and EG, thus avoiding the drawback of susceptibility to the alkalinity of the system. Moreover, the yield is high (300 mg/batch), resulting in the feasibility for separating the final product from the reaction system, which indicates good potential in large-scale production for industrial needs.

### The surface modification of the Fe_3_O_4_ nanoparticles with PAA

**Figure 2:**
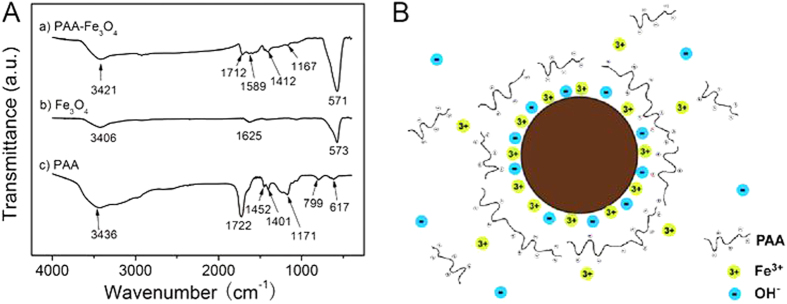
(**A**) IR spectra of a) PAA modified magnetic nanoparticles (FNPs), b) bare 100 nm FNPs, and c) PAA. (**B**) Schematic illustration of the PAA-modified FNPs dispersed in aqueous media.

The modification of FNPs with PAA was characterized by FT-IR spectrometer and Zeta potential measurement. Figure 2A demonstrates the FT-IR spectra of a) PAA-modified FNPs, b) bare 100 nm spherical FNPs and c) PAA. The peaks at 570 cm^−1^ in curve a and curve b confirm that the product is Fe_3_O_4_^29^. The C = O stretching vibration peak (1712 cm^−1^), C-O-C stretching vibration peak (1167 cm^−1^) and the characteristic absorption peaks of carboxylate at 1589 cm^−1^ and 1412 cm^−1^ in curve a confirm the successful chemisorption of PAA on the surface of the Fe_3_O_4_ particles. This result also indicates that part of the carboxyl groups on the short-chain PAA cooperate with Fe^3+^ cations on the surface of the particles while the rest part expose outward into the aqueous solution, conferring upon the particles a high degree of dispersibility in water^30^. The modification of FNPs with PAA was further confirmed by Zeta potential measurement. Due to the pH sensitivity of PAA, the Zeta potential of FNPs changes with pH, as shown in Figure S2.

The modification mechanism is schematically illustrated in Fig. 2B. The noncoordinated carboxylate groups on the PAA polymer chains extend into aqueous solution and render the particles surface highly charged. Therefore the modified FNPs are stabilized in aqueous media. The modification efficiency increases dramatically with adding the Fe^3+^ cations in the form of the PAA-Fe^3+^ solution and the modification time is obviously shortened. This is due to the preferential absorption of Fe^3+^ cations on the surface of the Fe_3_O_4_ and consequently a strong coordination is formed between the Fe^3+^ cations and the PAA, which makes free Fe^3+^ cations work as a bridge between the surface of the Fe_3_O_4_ and the PAA. This hypothesis could be supported by the experiment described as follows. When the PAA solution without the Fe^3+^ cations was added into the Fe_3_O_4_ precipitate (100 nm FNPs in this case), the dispersibility of the final product was poor with a modification time of 15 min. However, when the modification time was prolonged to 30 min, the mixed solution turned to orange and Fe^3+^ cations were released into the aqueous solution since the surface of the FNPs was corroded by PAA. Then the orange solution was collected and added into another unmodified Fe_3_O_4_ precipitate with a modification time of 15 min, and the dispersibility of the final product was better than those just treated with Fe^3+^-free PAA solution. Hence, just adding the Fe^3+^ cations in the PAA solution could effectively increase the modification efficiency.

### The stability of the PAA-modified Fe_3_O_4_ suspension

**Figure 3:**
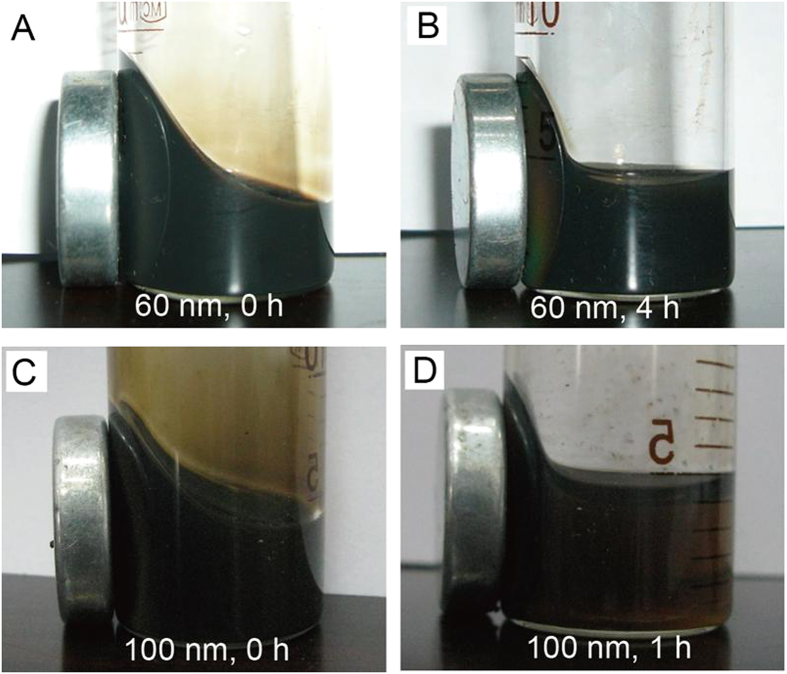
Time-dependent photographs of the 60 nm FNPs colloidal (*σ* = 120 *μ*S · cm^−1^) under electromagnetic field. (**A**) The photograph taken at the beginning of applying 2000 G magnetic field, (**B**) taken after 4-hour 2000 G magnetic field applied. (**C**) The photographs of the 100 nm particle suspension (*σ* = 250 *μ*S · cm^−1^) taken at the beginning of applying 2000 G magnetic field, and (**D**) taken after 1-hour 2000 G magnetic field applied.

The PAA-modified FNPs with excellent dispersibility in aqueous solution could tolerate relatively high ionic strength and remain stable even under strong magnetic field. The electrical conductivity is used to evaluate the ionic strength of the solution. The typical results of the stability test using 60 nm FNPs solution (electrical conductivity 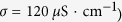 and 100 nm FNPs solution (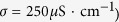 are given in Fig. 3. Even though the electrical conductivity of the particles solution is 20 times higher than that of the distilled water, it is difficult for 60 nm FNPs to precipitate completely after a 2000 G magnetic field applied for 4 h, which is shown in Fig. 3B. It can also be distinguished from Fig. 3D that, although the 100 nm FNPs precipitate faster than the 60 nm FNPs due to their larger size and mass, it still takes about 1 h to precipitate under a 2000 G magnetic field. Furthermore, the thermo-stability of Fe_3_O_4_ particles was investigated in Figure S3. The color pattern of the magnetochromatic photographs, as well as the peak location of the reflection spectra of Fe_3_O_4_ particles barely changed after heating the solution at 65 °C for 15, 30 and 45 min. The stability of the FNPs provided by the PAA significantly decreases the rate of aggregation and could give the particles enough time to form long-order structure in response to the external magnetic field.

### The magnetic properties of the PAA-modified Fe_3_O_4_ nanoparticles

**Figure 4:**
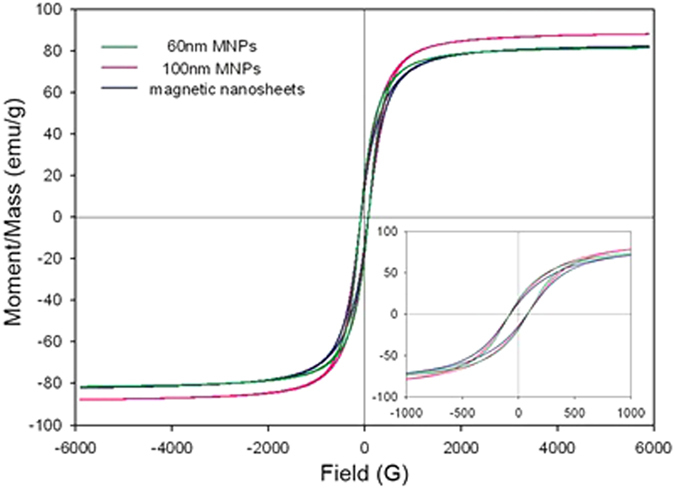
Magnetization curves of the modified FNPs with different sizes and shapes at room temperature. The inset shows the enlarged zone of the curves.

The magnetic properties of the modified particles and the corresponding solution were investigated respectively. As shown in Fig. 4, all the particles exhibit ferrimagnetism with low remanence and coercive force. The saturation magnetizations of 60 nm FNPs, 100 nm FNPs and magnetic nanosheets can be obtained to be 81.5, 88.0, 82.1 emu/g, respectively. Li *et al.* reported the high saturation magnetization of 81.9 emu/g for the as-prepared 200 nm Fe_3_O_4_ nanopheres^20^. Kolen’ko *et al.* recently obtained the 13~20 nm Fe_3_O_4_ nanoparticles with a saturation magnetization of 84.01 ± 0.25 emu/g^31^. The saturation magnetic values reported by Yin *et al.* were 63.5, 56.7, 30.9, and 21.2 emu/g respectively for the Fe_3_O_4_ particles with the size of 174, 93, 53 and 8 nm^16^. Compared with the reported data, the saturation magnetic value we reported is competitively high.

**Figure 5:**
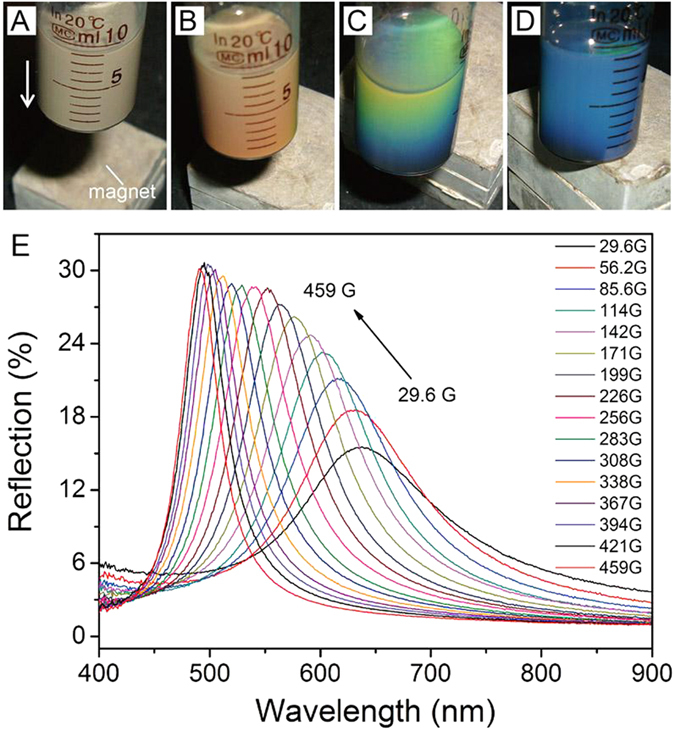
Magnetochromatic properties of Fe_3_O_4_ colloidal. (**A**–**D**) Photographs of particle suspension in response to different intensities of external magnetic field. The distance between the sample and the magnet decreases from (**A**–**D**). (**E**) Reflection spectra of particle suspension formed with 100 nm FNPs in response to external magnetic fields with various intensities.

Magnetization hysteresis loop is observed in the case of the particles solution as shown in Figure S4. The hysteresis loops of all the colloid solutions exhibit no remanence but display irreversibility when the applied external magnetic field is between 100 and 900 G. The enlarged part of the curve between 0 and 2000 G of a typical 100 nm FNPs solution is illustrated in Figure S5. Curve *a* and curve *b* are divided into three stages. In the first stage, when the external magnetic field is 0 G, the magnetic moments of FNPs are in random distribution due to the Brown motivation and the electrostatic interactions of the PAA. In the second stage, when the external magnetic field is between 0 and 900 G, the magnetic moments of FNPs partially align along the direction of the external magnetic field. In the third stage, the magnetic field is large enough that most of the magnetic moments align to the direction of the external magnetic field and the solution shows saturation magnetization. However, when the external field is first decreased from the third stage in curve *a*, the FNPs could still keep the saturation magnetization until the field is decreased to be about 700 G which is lower than the value of 900 G. And during the magnetic field is between 900 and 100 G, the magnetization value in curve *a* is clearly larger than that in curve *b*. This is due to the complex effect of metastable state of the magnetic dynamic nanoparticles and the possible relaxation characteristic of the formed chain-like structure when changing the external magnetic field, the Brownian dynamics of particles, and the electrostatic interactions due to the PAA. When the external magnetic field is decreased below 100 G, the Brownian motion and electrostatic interactions of PAA gradually exceed the total magnetic moments of the particles and disrupt the chain-like structure. At around 20 G the chain-like structure eventually disappears, which is consistent with the result obtained from the reflection spectra in Fig. 5E.

### The magnetochromatic properties of the modified Fe_3_O_4_ colloidal

When an external magnetic field is applied to the magnetic particles solution, the colloidal reflect visible light with certain wavelength. The photographs of 100 nm particles solution are taken and shown in Fig. 5A–D which response to the varying magnetic field by decreasing the distance between the magnet and the sample. Figure 5C shows clearly the gradient of reflection colors. The FNPs are well dispersed in water in the absence of an external magnetic field under a Brownian motion in Fig. 5A. When a magnetic field is applied, each nanoparticle is induced a magnetic dipole, of which the magnitude can be controlled by the strength of the applied field. As the magnetic field increases, the dipolar magnetic interaction energy of the particles increases and overcomes the thermal energy, the anisotropy of the attractive forces between induced dipoles causes particles a trend of aggregation. When the electrostatic repulsion interaction arises from the surfactant PAA of the particles and the magnetic attractive force reach a balance, the particles form linear chains structure along the direction of the external magnetic field with an interparticle distance^32^. When the magnetic field is decreased and smaller than the minimum field, the interaction force could not maintain the structure of the linear chains and the balance would be broken. The electrostatic interactions do not contribute to the ordering of the particles at this time and then the nanoparticles are randomly dispersed again.

The reflection spectra of particle (~100 nm) suspension at different intensities of external magnetic field from 29.6 to 459 G are shown in Fig. 5E. The suspension of particles with size larger than 100 nm could form one-dimension photonic crystal. The long-order structure formed by the particles reflects the visible light with different wavelengths which is dependent on the magnetic intensity. The reflection intensity increases and the reflection peaks blue shift as the magnetic field is increased from 29.6 to 459 G because the interparticle distance is decreased under a stronger magnetic field. The low reflection rate and broad peak width at low magnetic field are due to the Brownian motion, which significantly interferes with the formation of the long-range order structure. When the magnetic field is increased, the magnetic interaction between two Fe_3_O_4_ particles increases and surpasses the Brownian motion. The interparticle distance is decreased and the chain-like structure is therefore more compacted, which makes the reflection peak move to smaller wavelength. The degree of the blue-shift decreases when the magnetic field is larger than 400 G because of the strong repulsion of the hydrated layer of the absorbed PAA on the surface of Fe_3_O_4_ particles. Ge *et al.* demonstrated with experiments that by increasing the repulsive electrostatic interactions with increased number of washing cycles of residual reactant, their nanocrystals were organized with improved long-range order so that the diffraction intensity increased^33^. In our experiment, with increasing the external magnetic field, the increases of the intensity of reflection peaks are observed since the increased magnetic dipoles of the particles render the increasing of the magnetic attractive force and the long-range order is improved. The wavelength of the peak intensity provides a direct measure of the spacing between the nanoparticles. By using of the Bragg formula 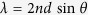 for 1D photonic crystal, where *λ* is the reflection wavelength, n is the reflection index of the particles solution, d is the separation between crystal planes and 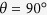 is the Bragg angle, the distance of two Fe_3_O_4_ particles along the direction of the magnetic field could be estimated. When the magnetic intensity is 459 G, *d* is calculated to be about 185 nm with the corresponding
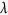
value in Fig. 5E. Since the diameter of Fe_3_O_4_ particles is confirmed to be 100 nm as shown in Fig. 1, the thickness of the hydrated layer is about 43 nm for each modified Fe_3_O_4_ particles.

### The visualization of magnetic field intensity on-chip by Fe_3_O_4_ colloidal

**Figure 6:**
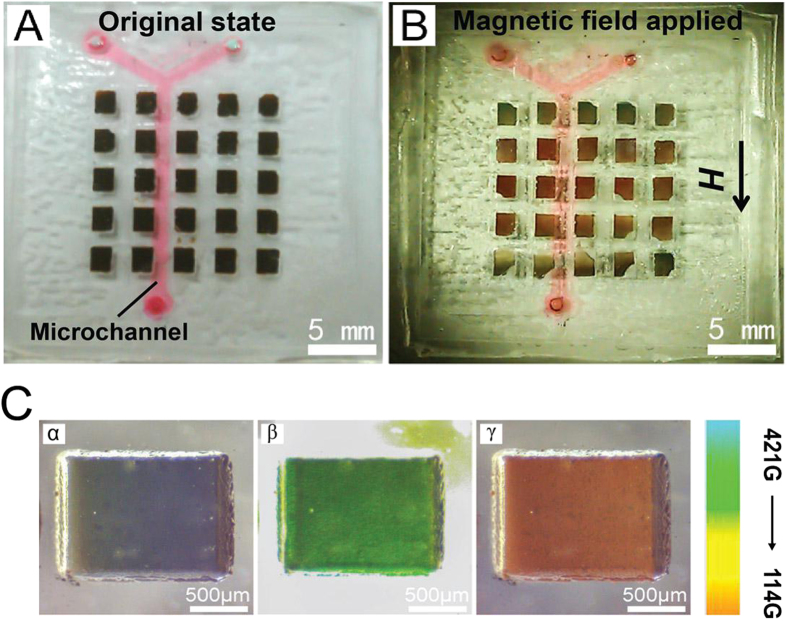
Visualization of the magnetic field distribution along a microfluidic channel on a chip. (**A**,**B**) are the photographs of the chip (**A**) before and (**B**) after the magnetic field was applied. The magnetochromatic Fe_3_O_4_ colloidal was sealed in the microsquares, and the microchannel is highlighted with red ink. (**C**) The colors of single sealed microsquare under different intensities of magnetic field. The color-intensity bar is showed in the right side.

As we known, conventional methods for measuring the magnetic field intensity only determine the properties over the whole volume of the sample^34^,^35^, the methods are limited to single-spot data acquisition and cannot provide local magnetization distribution in detail. Herein, we demonstrate the applications of this field responsive material for visualizing the magnetic field intensity on a chip by color changes. We incorporated this Fe_3_O_4_ suspension into microwells: a flat substrate filled with microwells of Fe_3_O_4_ colloidal (500-μm thick) is sealed with a PDMS microchannel to form a fluidic system (Fig. 6). The microwell determined the geometric dimensions of the color-tunable microstructure, and a permanent magnet was applied to generate an external magnetic field. In the absence of an external magnetic field, as shown in Fig. 6A, the sample in the microwells shows essentially no contrast. However, this color pattern changes its color from their initial appearance when an external magnetic field is applied along the microchannel. The intensity of the magnetic field can be estimated by comparing the color of the microwell with the color bar. The saturation magnetic values we measured are consistent with the results that measured by a commercial available magnetometer.

Hydrophilic Fe_3_O_4_ particles with controllable sizes and shapes have been successfully synthesized by a modified solvothermal approach followed by surface modification with short-chain PAA, which is easy-control and appropriate for mass production in industry. The carboxylate groups of PAA on the surface of FNPs extend into the aqueous solution, rendering the particle surfaces highly charged and hence the particles could be stabilized in aqueous media with excellent dispersibility and good thermal-stability. The saturation magnetization of the as-prepared 100 nm FNPs is 88.0 emu/g, which is probably the highest value reported. The suspension of particles could form one-dimension photonic crystal in response to external magnetic field ranging from 29.6 to 459 G, and reflection spectra cover the entire visible spectrum ranging from 480 ~ 680 nm. With the good magnetochromatic responses and high magnetic properties shown within the FNPs solution, the visualization of on-chip magnetic field intensity could be first realized and indicates good prospection. We believe that the simple synthetic strategy, as well as the excellent magnetic and magneto-optical properties of the as-prepared FNPs, would be of great interest to many researchers working in the fields of biomedical imaging, medical diagnostics and treatments, color display, and optical sensors, etc.

## Methods

### Synthesis of Fe_3_O_4_ particles with different sizes and shapes

The PAA-modified FNPs was prepared by a two-step method. Firstly, the FNPs were synthesized as follow. FeCl_3_·6H_2_O (1.03 g), FeCl_2_·4H_2_O (0.04 g), NaAc·3H_2_O (3.80 g), polyethylene glycol (PEG) (1.00 g, MW = 20000) were dissolved in a mixture of DEG and EG with different volume ratios of 26/14, 30/10 and 40/0 (total volume: 40 mL) under stirring and ultrasonic treatment. The homogeneous yellow mixture was then transferred into a Teflon-lined stainless-steel autoclave and sealed. After that, it was heated to 220 °C. It is worth noting that the inert gas protection is not necessary due to the closed system and the reducing environment created by EG and DEG. After reaction at 220 °C for 7 h, the autoclave was cooled down to room temperature. The as-obtained FNPs were washed several times with ethanol and DI water before the surface modification.

### Surface modification and dispersion of Fe_3_O_4_ particles

Secondly, the FNPs were modified with PAA as follow. PAA was synthesized by the method which has been described in detail in another paper^36^. 20 mL NaOH solution (1.0 mmol/L) was added into the wet Fe_3_O_4_ precipitation in a beaker, and then the mixture was ultrasonically treated to form FNPs suspension. 1 mL FeCl_3_ (0.1 mol/L) and 20 mL PAA (10.0 g/L) were previously mixed to form an orange solution. The FNPs suspension was slowly added into the orange solution under mechanical stirring. Afterwards, the mixture was ultrasonically treated at 80 °C for 15 min, and then the precipitate was collected by magnetic separation. After adding 20 mL NaOH solution (0.05 mol/L) into the precipitate and 10-s ultrasonic treatment of the mixture, the product was collected again by magnetic separation and washed by DI water for three times. Finally, the magnetic particle suspension was obtained by dispersing the product in a certain amount of DI water. It should be noted that the particles in the suspension are fairly stable in air and could be stored for months.

### Fabrication of microfluidic chips incorporating Fe_3_O_4_ suspension

PDMS mold with microwells and microchannel patterns were generated using standard soft lithography^37^. The chip was composed of three layers. The bottom microchannel layer and middle microwell layer were firstly bonded together to form an enclosed Y-shaped channel and uncovered microwells array. Then the Fe_3_O_4_ suspension was coated onto the PDMS mold to cover the whole area of the microwells. Extra material was scrapped off the PDMS surface with a glass slide. A flat PDMS mold (as the upper layer) was sealed to the PDMS mold with microwell array using semi-cured PDMS as adhesive, and the whole chip was formed.

### Characterization

Transmission electron microscopy (TEM) images were obtained on a high-resolution transmission electron microscope (HRTEM, JEM-2010) at an accelerating voltage of 200 kV. X-ray diffraction (XRD) patterns were recorded on a Power X-ray Diffractometer (D/Max-IIIA) with CuKα radiation. Infrared (IR) spectra were recorded with the wavenumbers ranging from 4000 to 500 cm^−1^ with a Nicolet model 759 Fourier transform infrared (FT-IR) spectrometer using a KBr wafer. Zeta potential was measured by using a Malvern Zetasizer Nano ZS90. The magnetic properties (M–H curves) were measured at room temperature by the use of a Lakeshore 7404-vsm magnetometer. The optical properties of the photonic crystal formed by the 100 nm FNPs under external magnetic field, which was controlled by a home-made electromagnet, were collected by using a QE65000-ABS Scientific-grade Optic Fiber Spectrometer (Ocean Optic, Inc.). The variation of the magnetic field which originates from the electromagnet was controlled by a DC electrometer. By changing the output current of the electrometer, the magnetic field was set ranging from 29.6 to 459 G and applied to the test tube containing the particles solution. Then the reflection spectra of the photonic crystal response to the varying magnetic field were recorded by putting the probe of the spectrometer onto the solution surface with a distance of 1 mm. The optical and microscope images of chips were taken by using an EOS 60D microscope digital camera (Canon) and a stereoscopic microscope (SZ760, Chongqing Optec Instrument Co., Ltd).

## Additional Information

**How to cite this article**: Zhuang, L. *et al.* Hydrophilic Magnetochromatic Nanoparticles with Controllable Sizes and Super-high Magnetization for Visualization of Magnetic Field Intensity. *Sci. Rep.* **5**, 17063; doi: 10.1038/srep17063 (2015).

## Supplementary Material

Supplementary Information

## References

[b1] Sun S., Murray C. B., Weller D., Folks L. & Moser A. Monodisperse FePt nanoparticles and ferromagnetic FePt nanocrystal superlattices. Science 287, 1989–1992 (2000). 10720318 10.1126/science.287.5460.1989

[b2] Miller M. M., Prinz G. A., Cheng S. F. & Bounnak S. Detection of a micron-sized magnetic sphere using a ring-shaped anisotropic magnetoresistance-based sensor: a model for a magnetoresistance-based biosensor. Appl. Phys. Lett. 81, 2211–2213 (2002).

[b3] Jain T. K., Morales M. A., Sahoo S. K., Leslie-Pelecky D. L. & Labhasetwar V. Iron oxide nanoparticles for sustained delivery of anticancer agents. Mol. Pharm. 2, 194–205 (2005). 15934780 10.1021/mp0500014

[b4] Babes L., Denizot B. T., Tanguy G., Le Jeune J. J. & Jallet P. Synthesis of iron oxide nanoparticles used as MRI contrast agents: a parametric study. J. Colloid Interface Sci. 212, 474–482 (1999). 10092379 10.1006/jcis.1998.6053

[b5] Chourpa I. *et al.* Molecular composition of iron oxide nanoparticles, precursors for magnetic drug targeting, as characterized by confocal raman microspectroscopy. Analyst 130, 1395–1403 (2005). 16172665 10.1039/b419004a

[b6] Kim J., Choi S.-E., Lee H. & Kwon S. Magnetochromatic microactuators for a micropixellated color-changing surface. Adv. Mater. 25, 1415–1419 (2013). 23299981 10.1002/adma.201203810

[b7] Kim J. *et al.* Real-time optofluidic synthesis of magnetochromatic microspheres for reversible structural color patterning. Small 7, 1163–1168 (2011). 21456096 10.1002/smll.201001822

[b8] Di Z., Chen X., Pu S., Hu X. & Xia Y. Magnetic-field-induced birefringence and particle agglomeration in magnetic fluids. Appl. Phys. Lett. 89, 211106 (2006).

[b9] Yusuf N. A., Ramadan A., Abu-Safia H. & Abu-Aljarayesh I. The curie-weiss behavior of birefringence in ferrofluids. J. Appl. Phys. 73, 6136–6138 (1993).

[b10] Pu S. *et al.* Measurement of the refractive index of a magnetic fluid by the retroreflection on the fiber-optic end face. Appl. Phys. Lett. 86, 171904 (2005).

[b11] Xu M. & Ridler P. J. Linear dichroism and birefringence effects in magnetic fluids. J. Appl. Phys. 82, 326–332 (1997).

[b12] Kooij E. S., Gâlcă A. C. & Poelsema B. Versatile transmission ellipsometry to study linear ferrofluid magneto-optics. J Colloid Interface Sci. 304, 261–270 (2006). 16997315 10.1016/j.jcis.2006.08.062

[b13] Horng H.-E., Yang S. Y., Lee S. L., Hong C. Y. & Yang H. C. Magnetochromatics of the magnetic fluid film under a dynamic magnetic field. Appl. Phys. Lett. 79, 350–352 (2001).

[b14] Nakatsuka K., Jeyadevan B. & Neveu S. The magnetic fluid for heat transfer applications. J. Magn. Magn. Mater. 252, 360–362 (2002).

[b15] Si S. *et al.* Size-controlled synthesis of magnetite nanoparticles in the presence of polyelectrolytes. Chem. Mater. 16, 3489–3496 (2004).

[b16] Ge J., Hu Y., Biasini M., Beyermann W. P. & Yin Y. Superparamagnetic magnetite colloidal nanocrystal clusters. Angew. Chem., Int. Ed. 46, 4342–4345 (2007). 10.1002/anie.200700197 17465432

[b17] Guan N. N., Xu J., Wang L. Y. & Sun D. J. One-step synthesis of amine-functionalized thermo-responsive magnetite nanoparticles and single-crystal hollow structures. Colloids Surf. A 346, 221–228 (2009).

[b18] Han C. L., Cai W. P., Tang W., Wang G. Z. & Liang C. H. Protein assisted hydrothermal synthesis of ultrafine magnetite nanoparticle built-porous oriented fibers and their structurally enhanced adsorption to toxic chemicals in solution. J. Mater. Chem. 21, 11188–11196 (2011).

[b19] Xiong Y., Ye J., Gu X. Y. & Chen Q. W. Synthesis and assembly of magnetite nanocubes into flux-closure rings. J. Phys. Chem. C 111, 6998–7003 (2007).

[b20] Deng H. *et al.* Monodisperse magnetic single-crystal ferrite microspheres. Angew. Chem., Int. Ed. 44, 2782–2785 (2005). 10.1002/anie.200462551 15798982

[b21] Wang L. Y., Yang Z. H., Zhang Y. & Wang L. Bifunctional nanoparticles with magnetization and luminescence. J. Phys. Chem. C 113, 3955–3959 (2009).

[b22] Vijayakumar R., Koltypin Y., Felner I. & Gedanken A. Sonochemical synthesis and characterization of pure nanometer-sized Fe_3_O_4_ particles. Mater. Sci. Eng. A 286, 101–105 (2000).

[b23] Hyeon T., Lee S. S., Park J., Chung Y. & Na H. B. Synthesis of highly crystalline and monodisperse maghemite nanocrystallites without a size-selection process. J. Am. Chem. Soc. 123, 12798–12801 (2001). 11749537 10.1021/ja016812s

[b24] Hui C. *et al.* Large-scale Fe_3_O_4_ nanoparticles soluble in water synthesized by a facile method. J. Phys. Chem. C 112, 11336–11339 (2008).

[b25] Kahl S., Grishin A. M., Khartsev S. I., Kawano K. & Abell J. S. Bi_3_Fe_5_O_12_ thin film visualizer. IEEE Trans. Magn. 3, 2457–2459 (2001).

[b26] Klank M., Hagedorn O., Shamonin M. & Dotsch H. Sensitive magneto-optical sensors for visualization of magnetic fields using garnet films of specific orientations. J. Appl. Phys. 92, 6484–6488 (2002).

[b27] Iwahara M. & Yamada S. Visualization of magnetic field by means of the projection method with signed objectives. J. Magn. Magn. Mater. 272–276, 2263–2265 (2004).

[b28] Lin Z. W., Zhu J. G., Guo Y. G., Zhong J. J. & Yu W. Y. Visualization of magnetic flux distribution at soft magnetic composite. Int. J. Appl. Electrom. 15, S25–S28 (2007).

[b29] Zaitsev V. S., Filimonov D. S., Presnyakov I. A., Gambino R. J. & Chu B. Physical and chemical properties of magnetite and magnetite-polymer nanoparticles and their colloidal dispersions. J. Colloid Interface Sci. 212, 49–57 (1999). 10072274 10.1006/jcis.1998.5993

[b30] Lopes I., Piao L., Stievano L. & Lambert J.-F. Adsorption of amino acids on oxide supports: a solid-state NMR study of glycine adsorption on silica and alumina. J. Phys. Chem. C 113, 18163–18172 (2009).

[b31] Kolen’ko Y. V. *et al.* Large-scale synthesis of colloidal Fe_3_O_4_ nanoparticles exhibiting high heating efficiency in magnetic hyperthermia. J. Phys. Chem. C 118, 8691–8701 (2014).

[b32] Liu J. *et al.* Field-induced structures in ferrofluid emulsions. Phys. Rev. Lett. 74, 2828–2831 (1995). 10058028 10.1103/PhysRevLett.74.2828

[b33] Ge J., Hu Y., Zhang T., Huynh T. & Yin Y. Self-assembly and field-responsive optical diffractions of superparamagnetic colloids. Langmuir, 24, 3671–3680 (2008). 18269296 10.1021/la7039493

[b34] Zhu J. G., Zhong J. J., Ramsden V. S. & Guo Y. G. Power losses of soft magnetic composite materials under two-dimensional excitation. J. Appl. Phys. 85, 4403–4405 (1999).

[b35] Lin Z. W., Zhu J. G., Guo Y. G., Wang X. L. & Ding S. Y. Three-dimensional hysteresis of soft magnetic composite. J. Appl. Phys. 99, 08D909 (2006).

[b36] Zhuang L. *et al.* Preparation and characterization of Fe_3_O_4_ particles with novel nanosheets morphology and magnetochromatic property by a modified solvothermal method. Sci. Rep. 5, 9320 (2015). 25799320 10.1038/srep09320 PMC4369971

[b37] Zhou J., Yan H., Zheng Y. & Wu H. Highly fluorescent poly(dimethylsiloxane) for on-chip temperature measurements. Adv. Funct. Mater. 19, 324–329 (2009).

