# Supplementary material for: Hydrophilic Magnetochromatic Nanoparticles with Controllable Sizes and Super-high Magnetization for Visualization of Magnetic Field Intensity
Source: Sci Rep. 2015 Nov 23;5:17063. doi: 10.1038/srep17063 (PMC4655409; doi:10.1038/srep17063)
Supplement: Supplementary Information [file srep17063-s1.doc]

Supplementary information for

Hydrophilic Magnetochromatic Nanoparticles with Controllable Sizes and Super-high Magnetization for Visualization of Magnetic Field Intensity

Lin Zhuang^1^, Yongxin Zhao^1^, Huixiang Zhong^2^, Jinhua Liang^1^, Jianhua Zhou^2^ & Hui Shen^1^

^1^ School of Physics and Engineering, State Key Laboratory of Optoelectronic Materials and Technologies, Sun Yat-sen University, Guangzhou 510006, China

^2^ Key Laboratory of Sensing Technology and Biomedical Instruments of Guangdong Province, School of Engineering, Sun Yat-sen University, Guangzhou 510006, China

Corresponding authors:

Tel: +862039387890;

Fax: +862039387890;

E-mail address: zhoujh33@mail.sysu.edu.cn (J. H. Zhou)


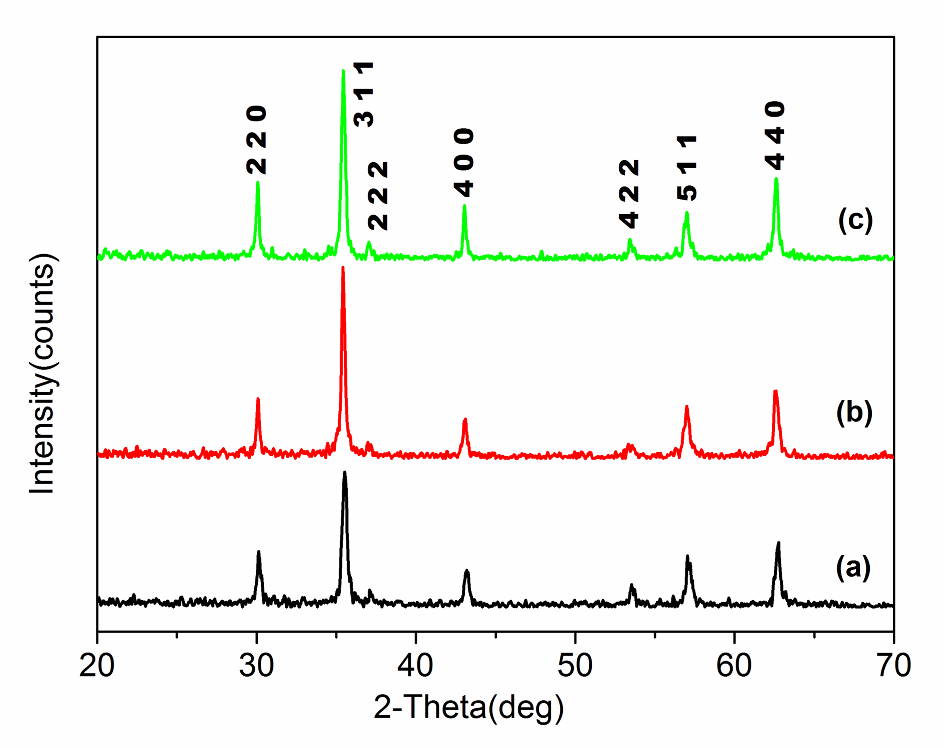


**Figure S1.** XRD patterns of (a) 60nm Fe_3_O_4_ spherical particles synthesized with DEG/EG=30/10, (b) 100 nm Fe_3_O_4_ spherical particles synthesized with DEG/ EG=26/14, and (c) Fe_3_O_4_ nanosheets synthesized with DEG/EG=40/0.

The X-ray diffraction patterns of the as-prepared Fe_3_O_4_ nanoparticles and nanosheets synthesized with different DEG/EG volume ratios are shown in Figure 1S, which reveal the formation of magnetite iron oxide with well-defined crystallinity via the modified solvothermal synthesis process. The XRD patterns depict Fe_3_O_4_ with seven characteristic peaks corresponding to the crystal planes of (220), (311), (222), (400), (422), (511) and (440) respectively, which is consistent with the standard data for the inverse spinel Fe_3_O_4_ and cubic phase according to the reported data (JCPDS card no.65-3107). No obvious XRD peaks arising from impurities are found. There is no evidence that Fe_3_O_4_ particles are contaminated by foreign materials in the system.


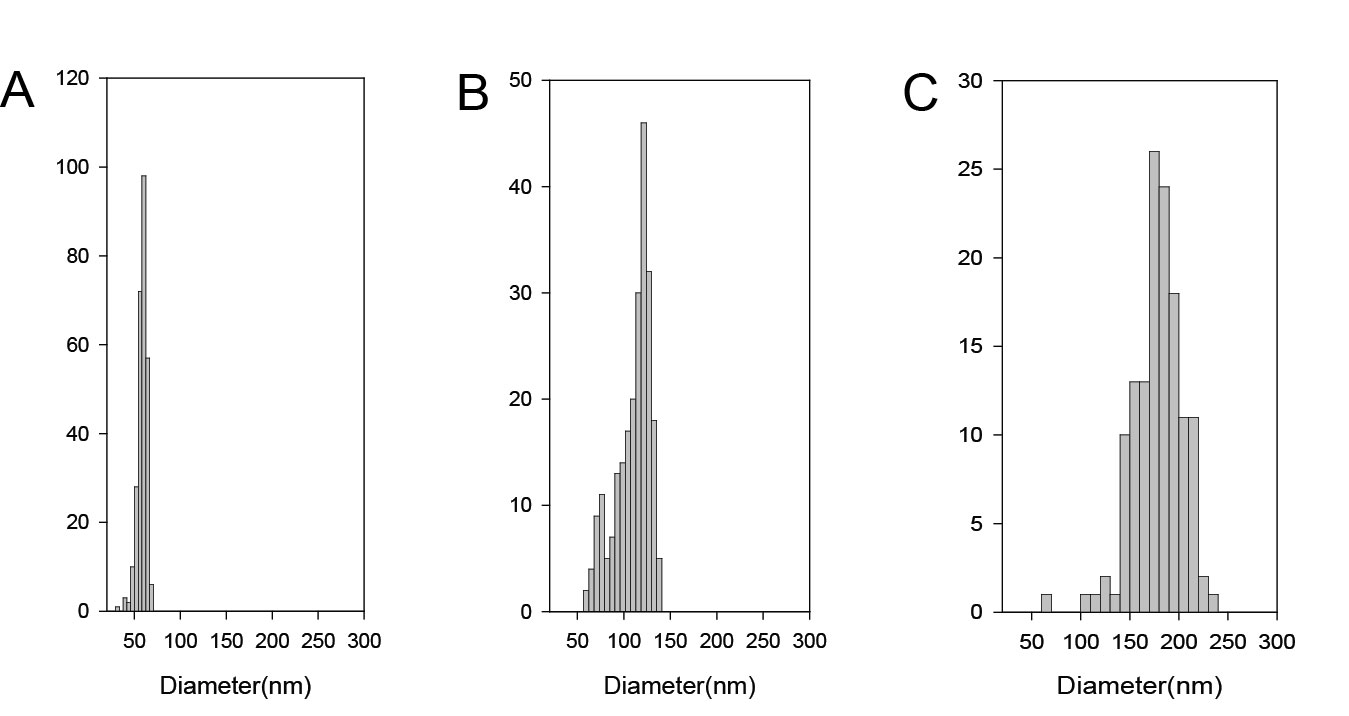


**Figure S2.** The size distribution of the fabricated Fe_3_O_4_ spherical nanoparticles in Figure 1: (a) 60 nm; *ɛ* = 0.08; (B) 100 nm; *ɛ* = 0.16; (C) 180 nm; *ɛ* = 0.14, *ɛ* is the distribution coefficient and the ε values are relatively low for our nanoparticles.


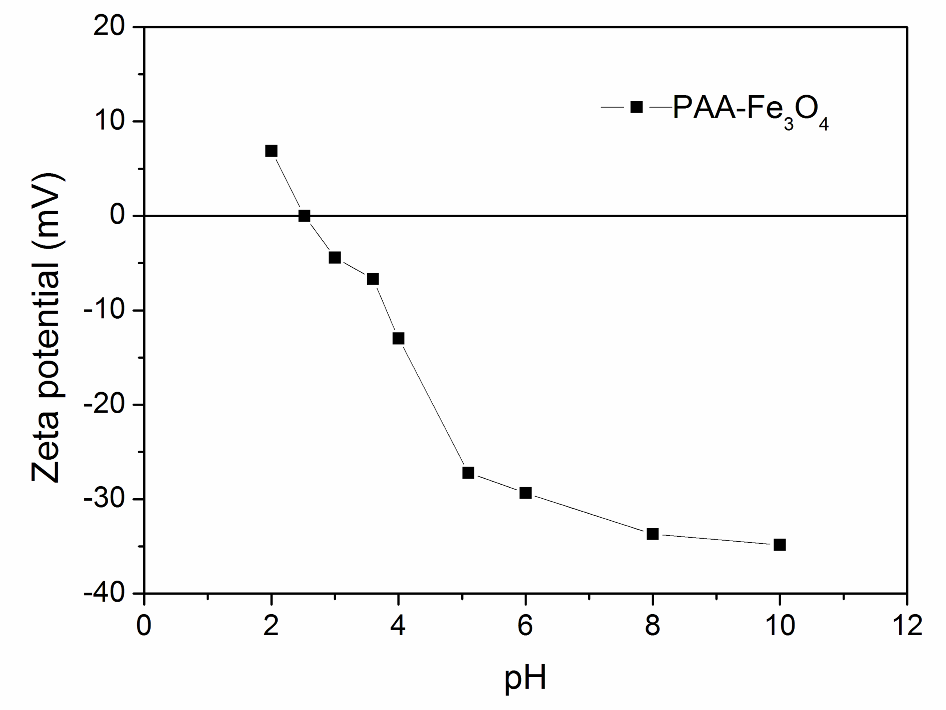


**Figure S3.** Zeta potential of the PAA-Fe_3_O_4_ nanoparticles at various pH values.

Different from Yin's method^16^, the surface modification of the FNPs in our method was performed after the synthesis process of the particles (*i.e.* the second step). The stabilizer is not involved in the synthesis process of magnetic particles, which simplifies the synthesis process and increases the reproducibility. Therefore, the FNPs could be functionalized with other materials (*e.g.* amino acid^[1]^ and quantum dots) during the second step (see Figure S2-X).

[1] Y. Xu, L. Zhuang, et.al., Preparation and characterization of polyacrylic acid coated magnetite nanoparticles functionalized with amino acids, Thin Solid Films 544 (2013) 368-373. (The paper of our group has been published).

We performed the experiment that bare Fe_3_O_4_ particles were modified with C quantum dots. The fluorescence microscope photograph in Figure S2-X confirms that the quantum dots have been linked on the particles surface successfully, which indicates well that the FNPs prepared by our method could be functionalized with other materials including quantum dots.


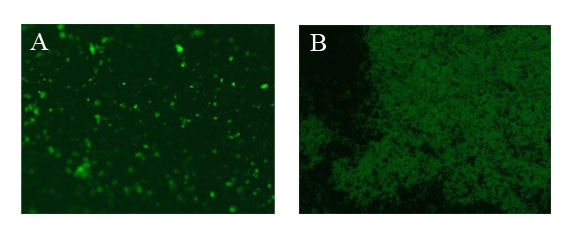


**Figure S3-X.** The fluorescence microscope photographs of Fe_3_O_4_ nanoparticles linked with quantum dots. (A) and (B) are photographs with different resolution.


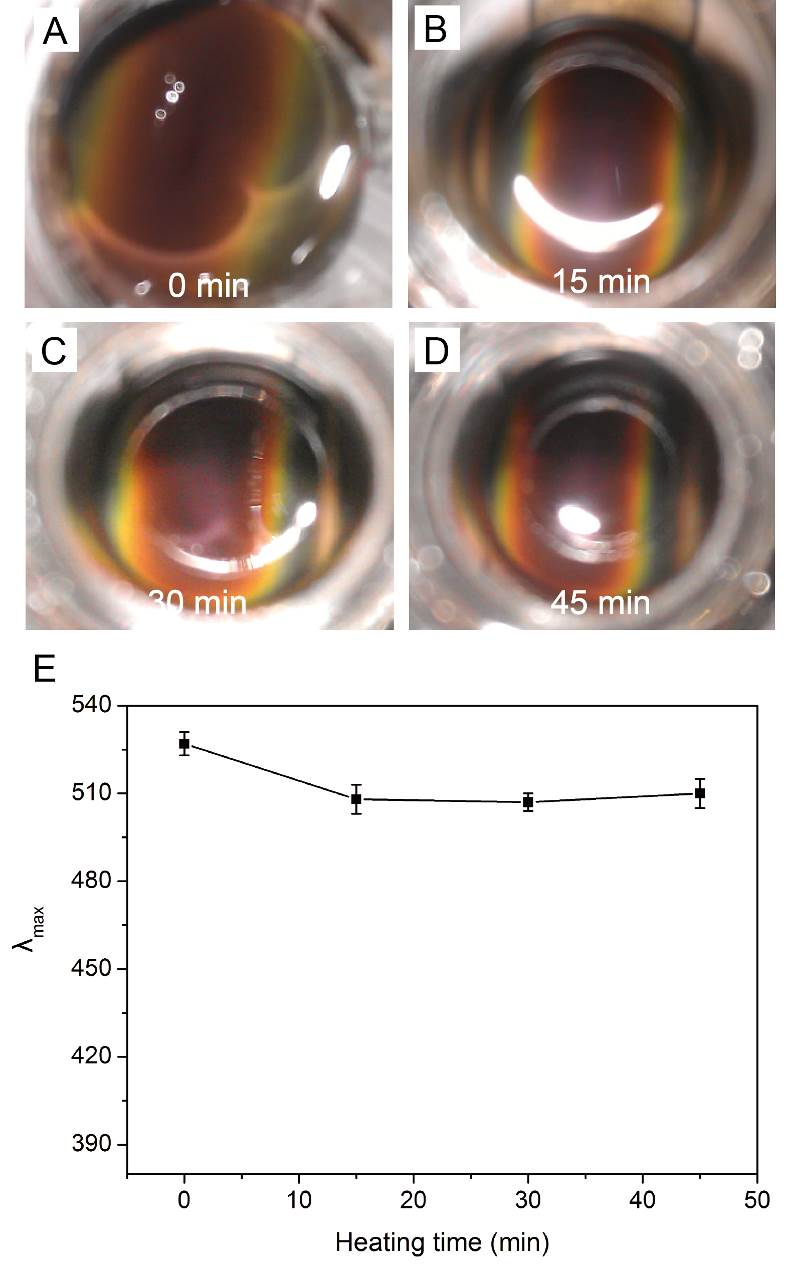


**Figure S4.** The thermo-stability of the PAA-Fe_3_O_4_ colloidal at 65 ^o^C. (A)🡪(D) are the photographs of particle suspension at different time. (E) Time-dependent peak location of reflection spectra.


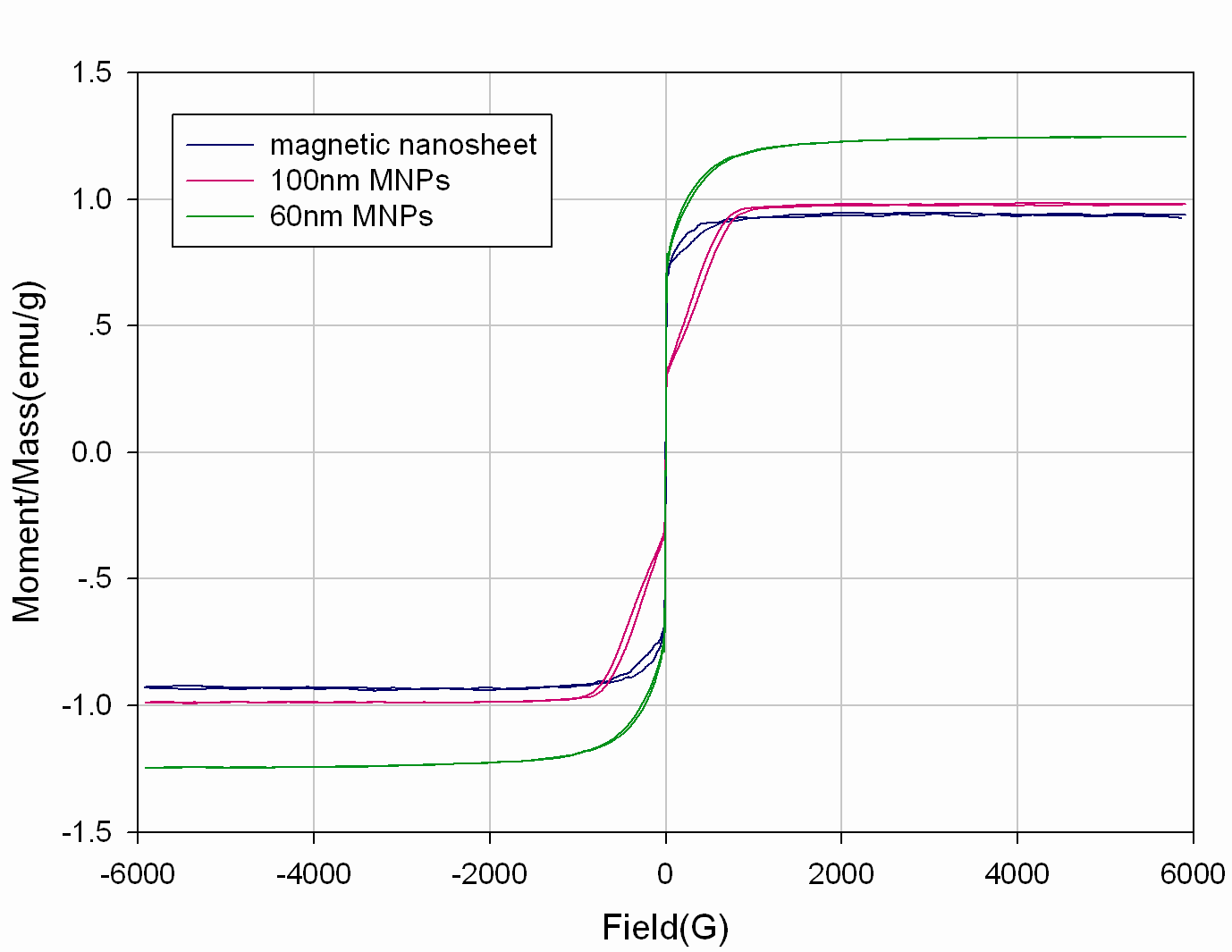


**Figure S5.** Magnetization curves of particles suspension with different sizes and shapes of the FNPs at room temperature.


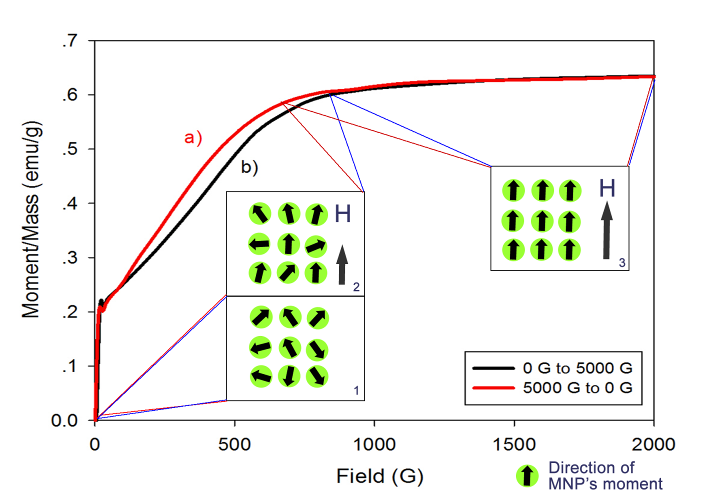


**Figure S6.** Schematic illustration of the magnetization curve of the 100 nm MNPs suspension. Curve a is the magnetization curve when the magnetic field is decreased, while curve b is the one when the magnetic field is increased.


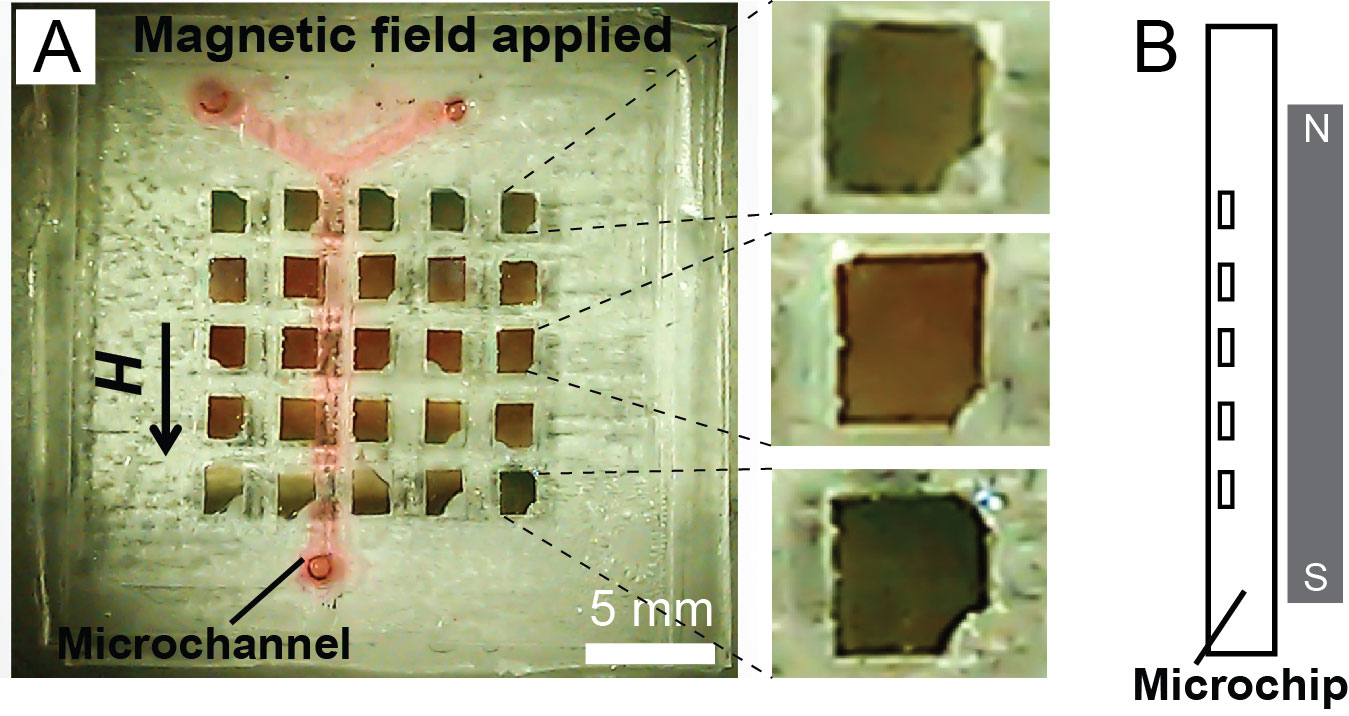


**Figure S7.** (A) The photographs of the chip and the enlarged view of the microwells at different locations of microchip after the magnetic field was applied. (B) A schematics showing the assembly of microchip and the magnet.
